# CCL2 Overexpression in the Brain Promotes Glial Activation and Accelerates Tau Pathology in a Mouse Model of Tauopathy

**DOI:** 10.3389/fimmu.2020.00997

**Published:** 2020-05-20

**Authors:** Aurelie Joly-Amado, Jordan Hunter, Zainuddin Quadri, Frank Zamudio, Patricia V. Rocha-Rangel, Deanna Chan, Anisha Kesarwani, Kevin Nash, Daniel C. Lee, Dave Morgan, Marcia N. Gordon, Maj-Linda B. Selenica

**Affiliations:** ^1^Molecular Pharmacology and Physiology, College of Medicine, University of South Florida, Tampa, FL, United States; ^2^Pharmaceutical Sciences, College of Pharmacy, University of South Florida, Tampa, FL, United States; ^3^Michigan State University, Department of Translational Neuroscience, Grand Rapids, MI, United States; ^4^Sanders-Brown Center on Aging, Department of Molecular and Cellular Biochemistry, University of Kentucky, Lexington, KY, United States

**Keywords:** monocyte chemoattractant protein-1 (MCP-1), neuroinflammation, Alzheimer's disease, tau, Aβ, gene therapy

## Abstract

Innate immune activation is a major contributor to Alzheimer's Disease (AD) pathophysiology, although the mechanisms involved are poorly understood. Chemokine C-C motif ligand (CCL) 2 is produced by neurons and glial cells and is upregulated in the AD brain. Transgene expression of CCL2 in mouse models of amyloidosis produces microglia-induced amyloid β oligomerization, a strong indication of the role of these activation pathways in the amyloidogenic processes of AD. We have previously shown that CCL2 polarizes microglia in wild type mice. However, how CCL2 signaling contributes to tau pathogenesis remains unknown. To address this question, CCL2 was delivered via recombinant adeno-associated virus serotype 9 into both cortex and hippocampus of a mouse model with tau pathology (rTg4510). We report that CCL2 overexpression aggravated tau pathology in rTg4510 as shown by the increase in Gallyas stained neurofibrillary tangles as well as phosphorylated tau-positive inclusions. In addition, biochemical analysis showed a reduction in the levels of detergent-soluble tau species followed by increase in the insoluble fraction, indicating a shift toward larger tau aggregates. Indeed, increased levels of high molecular weight species of phosphorylated tau were found in the mice injected with CCL2. We also report that worsening of tau pathology following CCL2 overexpression was accompanied by a distinct inflammatory response. We report an increase in leukocyte common antigen (CD45) and Cluster of differentiation 68 (CD68) expression in the brain of rTg4510 mice without altering the expression levels of a cell-surface protein Transmembrane Protein 119 (Tmem119) and ionized calcium-binding adaptor molecule 1 (Iba-1) in resident microglia. Furthermore, the analysis of cytokines in brain extract showed a significant increase in interleukin (IL)-6 and CCL3, while CCL5 levels were decreased in CCL2 mice. No changes were observed in IL-1α, IL-1β, TNF-α. IL-4, Vascular endothelial growth factor-VEGF, IL-13 and CCL11. Taken together our data report for the first time that overexpression of CCL2 promotes the increase of pathogenic tau species and is associated with glial neuroinflammatory changes that are deleterious. We propose that these events may contribute to the pathogenesis of Alzheimer's disease and other tauopathies.

## Introduction

Alzheimer's disease (AD) is a progressive neurodegenerative disease, which is characterized by the formation of extracellular amyloid plaques (or senile plaques) and intracellular neurofibrillary tangles resulting from tau protein hyperphosphorylation and aggregation. Glial and innate immune activation are also hallmarks of AD although their contribution to the etiopathology of the disease is unclear. Microglia, the resident inflammatory cells of the brain, are found in a highly activated state in the AD brain. Activation of microglia is indicated by morphological alterations, proliferation, increased expression of cell surface receptors, and secretion of cytokines and chemokines (1). In AD, Aβ and tau pathology have been shown to activate microglia both *in vivo* and *in vitro* (2). Interestingly, microglial activation can precede the emergence of amyloid or tau pathology in some mouse models (3, 4), suggesting that it is an early event promoting Aβ and tau pathologies. The CC-chemokine ligand 2 (CCL2), also known as monocyte chemotactic protein-1 (MCP-1), is present in the brain and produced by microglia, neurons, activated astrocytes, and mononuclear phagocytes (5). CCL2 binds to the CC-chemokine receptor 2 (CCR2) to regulate cell infiltration into peripheral tissue and brain during infectious and inflammatory events affecting disease processes (6–8). Data analysis of cytokines and chemokines levels in brain tissue from AD patients revealed an increase in CCL2 expression compared to age matched healthy patients (9, 10). Interestingly, in brain tissue of AD patients, CCL2 is present in neurons, astrocytes, reactive microglia, as well as senile plaques and micro vessels (9–12). Further, CCL2 levels in CSF (13) and plasma (14) correlates with a faster cognitive decline in AD patients and in an asymptomatic aging adult population (15). Thus, CCL2 seems to be a viable candidate to glial activation in the neuropathology of AD and other tauopathies.

Studies of CCL2 in animal models with amyloid deposition have highlighted the role of CCL2 in the disease and its contribution to AD pathology. In particular, it appears that CCL2-signaling can exacerbate Aβ pathology in animal models of AD. For instance, Yamamoto and colleagues have demonstrated that the bigenic APP/CCL2 mice, overexpressing CCL2 under the control of the human glial fibrillar acidic protein (GFAP) promoter, displayed increased microgliosis and astrogliosis, enhanced Aβ aggregation and amyloid plaques with no alteration of APP processing when compared to APP mice (16). The authors later reported hippocampal synaptic dysfunction and worsening of memory impairment in this model (17). Conversely, double mutant APP/PS1/CCL2 null mice also displayed increased levels of Aβ oligomers, microglia accumulation around plaques, impaired neurogenesis and worsening of cognitive dysfunction (18). Similarly, a total deficiency in CCR2 precipitates Aβ accumulation by decreasing Aβ clearance in APP mice (19) demonstrating the ambiguity of the role of CCL2 on Aβ pathology.

Opposite results were however observed in a recent study showing that total CCL2 deficiency in the 5xFAD amyloid mouse model, improved cognition during Y-maze, normalized IL-1β and CCL3 levels, reduced astrogliosis and amyloid plaque formation as well as Aβ accumulation and neurodegeneration (20). In parallel, reduction of microglia activation and Aβ accumulation in APP/PS1 following genetic manipulations correlated with decreased CCL2 in the brain (21) consistent with Gutierrez's findings. Although the mechanisms of action of this dual role for CCL2 remain to be elucidated, it appears clear that CCL2—CCR2 signaling contributes to Aβ pathology, independently of an APP processing mechanism. However, the studies investigating the role of CCL2—CCR2 pathway in tau pathology are very scarce. Indeed, we could only find one report relating the effects of CCR2 manipulation on tau pathology in which genetic deletion of Ccr2 reduced traumatic lesions but enhanced endogenous tau hyperphosphorylation in the brain of a mouse model of traumatic brain injury (22). This emphasizes the need for further studies elucidating the role of CCL2 in tau pathology.

Toward this goal, we investigated the role of CCL2 cerebral overexpression on glial activation and tau pathology in the rTg4510 mouse model of tauopathy. We have previously shown that overexpression of CCL2 through rAAV9 transduction achieved glial activation in aged non-transgenic mice (23). Therefore, overexpression of CCL2 or RFP via rAAV9-virus was utilized to transduce anterior cerebral cortex and hippocampus of 5-month-old rTg4510 tau mice. We report that CCL2 overexpression induced worsening in tau pathology by inducing tau phosphorylation, increase in phosphorylated high molecular tau species as well as insoluble tau. We also found that CCL2 induced microgliosis and changes in astrocytic morphogenesis together with changes in inflammatory cytokines in the brain of rTg4510 mice.

Altogether our data show for the first time that cerebral overexpression of CCL2 facilitates tau accumulation together with glial neuroinflammatory changes in a relevant mouse model of tauopathy. Neuroinflammatory pathways and specifically the CCL2—CCR2 axis could be determinant factors in identifying new targets that aim at reducing the common underlying pathology in Alzheimer's disease and dementia-related tauopathies.

## Methods

### Animals

All animal testing procedures were approved by the Institutional Animal Care and Use Committee of the University of South Florida and were performed in accordance with the eighth edition of the “Guide for the Care and Use of Laboratory Animals,” published by the National Academy of Science, the National Academies Press, Washington, DC (2011).

The regulatable Tg4510 (rTg4510) mouse line was used for this study. Parental mutant tau and tetracycline-controlled transactivator (tTA) protein mouse lines were maintained separately and bred to produce rTg4510, non-transgenic and tTA only littermates as described previously (24). The rTg4510 mouse carries the P301L mutation (tetO-MAPT^*^P301L), which is associated with an autosomal dominantly inherited dementia referred to as frontotemporal dementia and parkinsonism linked to chromosome 17 (FTDP-17) (25). Although recent work suggests that some of the Tg4510 phenotype may be due to insertional mutagenesis events (26), this should not confound comparisons of treatments within the rTg4510 line.

Transgenic APP/PS1 mice were obtained from a breeding colony at the University of South Florida and have been maintained for >20 years. This line has been shown to have a selective increase in the level of Aβ42 as opposed to Aβ40 (27) and to develop extracellular Aβ deposits, which increase in number, size and density with aging (28). Mice were housed individually and maintained on a 12-h light/dark cycle. Food (E2018, Envigo) and water was given *ad libitum*

### Experimental Design

Five-month-old rTg4510 mice balanced for sex underwent intracranial bilateral injection of rAAV9-CCL2 (*n* = 6) or red fluorescent protein (RFP, *n* = 6) in both the hippocampus and the anterior cortex for a total of four injections. Two months after the intracranial injections, brain tissue was collected.

To measure the effect of CCL2 overexpression on amyloid pathology, a similar experiment was conducted on APP/PS1 mice. Twenty-month-old APP/PS1 mice received intracranial bilateral injection of rAAV9-CCL2 (*n* = 10) or green fluorescent protein (GFP, *n* = 10) in both the hippocampus and the anterior cortex. Four months after intracranial injections, brain tissue was collected.

For all animals and post-perfusion, half of the brain was collected for histology and tissue from the other half of the brain was dissected and utilized for western analysis or multiplex assays as described below. Studies conducted at our laboratory for almost one decade have demonstrated the efficacy of recombinant adeno-associated virus (rAAV) serotype 9 to transduce neurons within specific regions in the mouse brain (29–32). rAAV9 drives robust gene expression as early as 1 week *in vitro* and *in vivo* (33, 34) and persists for more than 9 months in mouse brain (35). Therefore, rAAV9 is a preferred candidate to deliver CCL2/RFP *in vivo* as previously reported (23). We chose to examine neuroinflammation and tau pathology after 2–4 months of viral injection to allow strong expression and to provide time for recovery from the intracranial injection.

### CCL2/RFP-AAV9 Preparation

The CCL2/RFP clone was generated as previously described (23). Briefly, mouse cDNA was amplified using PCR with primers 5′- GAGACCGGTCCACCATGCAGGTCCCTGTCATGCTTC-3′ and 5′GAGGCTAGCCTAGTTCACTGTCACACTGGTCACTCC-3′. CCL2 was cloned into the AgeI and NheI sites of the rAAV vector pTR2-RMCS under the control of the hybrid cytomegalovirus chicken β-actin promoter. This vector also expresses red fluorescent protein (RFP) under the control of the thymidine kinase promoter and AAV2 terminal repeats. rAAV serotype 9 virus was generated using pAAV9 and pXX6 in HEK293 cells as described previously (23, 29).

### Intracranial Injections

The injection procedures were performed using a convection-enhanced delivery method described previously (29). Briefly, mice were anesthetized with 1.5% isoflurane with oxygenation and secured into a stereotactic apparatus. Five-mo.-old rTg4510 mice received bilateral injections of 2 μl of rAAV9-CCL2 (7 × 10^∧^12 vector genomes (vg)/ml, *n* = 6) or 2 μl of rAAV9-RFP (control, *n* = 6 × 10^∧^12 vg/ml, control, *n* = 6) into both cortex and hippocampus. Twenty-mo.-old APP/PS1 mice received bilateral injections of 2 μl of rAAV9-CCL2 (7 × 10^∧^12 vector genomes (vg)/ml, *n* = 6) or 2 μl of rAAV9-GFP (control, *n* = 9 × 10^∧^12 vg/ml, control, *n* = 6). The coordinates of injection were as follows: hippocampus (from bregma) anteroposterior −2.7 mm, lateral ±2.7 mm, dorsoventral −3.0 mm; cortex, anteroposterior (from bregma) +2.2 mm, lateral ±1.7 mm, dorsoventral −3.0 mm. A microsyringe injector and controller (Stoelting, Wood Dale, IL, USA) were used to inject 2 μl of the virus at a constant rate of 2.5 μl/min in each placement. The needle was kept in place for 1 min after injection and then raised slowly. Mice were allowed to recover for 2 months.

### Tissue Collection

Mice were euthanized 2 (rTg4510 mice) or 4 (APP/PS1) months after injection, at 7 or 24 months of age, with a solution containing pentobarbital and phenytoin, then transcardially perfused with 25 ml of 0.9% normal saline solution. Brains were collected immediately following perfusion. One hemisphere was dissected and frozen for western blot analysis and the second hemisphere was immersion fixed in 4% phosphate-buffered paraformaldehyde for 24 h. The fixed hemispheres were cryoprotected in successive incubations of 10, 20, and 30% sucrose solutions for 24 h each. Subsequently, brains were frozen on a cold stage and sectioned in the horizontal plan (25 μm thickness) on a sliding microtome and stored in Dulbecco's phosphate buffered saline with 10 mM sodium azide solution at 4°C. Keeping one hemisphere for biochemistry and one for histochemistry allows to perform both analyses on all the animals (*N* = 6 per treatment group for western blot as well as immunochemistry).

### Histopathology

Stereological principles were employed to select the sections stained for each marker. Immunohistochemical procedural methods were described (28). Six to eight sections per animal (*N* = 6 per treatment group) were placed in a multi-sample staining tray and endogenous peroxidase was blocked (10% methanol, 3% H_2_O_2_ in phosphate buffered saline, 10 mM NaPO4, 0.8%NaCl, pH 7.4, PBS;30 min). Tissue samples were permeabilized with 0.2% lysine, 1%Triton X-100 in PBS solution and incubated overnight in primary antibody. The following primary antibodies were used for immunohistochemistry: CCL2 (rat anti-CCL2; R&D Systems, Minneapolis, MN, USA), CD45 (rat anti-mouse; AbD Serotec, Raleigh, NC, USA), CD68 (Bio-Rad, Hercules, CA), Iba-1 (Wako, Richmond, VA) anti-tau phosphorylated at Ser396 (rabbit polyclonal, Anaspec, Fremont, CA), total tau H150 (rabbit polyclonal, SantaCruz Biotechnology), anti-tau phosphorylated at Ser202/Thr205 AT8 (Thermo scientific, Waltham, MA), anti-Aβ (prepared by Paul Gottschall, UAMS, Arkansas, USA). Sections were washed in PBS, and then incubated in corresponding biotinylated secondary antibody (Vector Laboratories, Burlingame, CA). The tissue was again washed after 2 h and incubated with Vectastain® Elite® ABC kit (Vector Laboratories, Burlingame, CA) for enzyme conjugation. Finally, sections were stained using 0.05% diaminobenzidine, 0.5% nickel ammonium sulfate and 0.03% H_2_O_2_. Tissue sections were mounted onto slides, dehydrated, cover slipped and prepared for analysis.

Immunofluorescence labeling for GFAP (Abcam, Burlingame, CA), Tmem119 (rabbit polyclonal, abcam, Cambridge, UK) and Iba-1 (goat polyclonal, abcam, Cambridge, UK) was performed as follows. After incubation with the primary antibody, the free-floating sections were incubated for 2 h with corresponding Alexa Fluor 488 or 647 fluorophore-coupled antibody (1:1,500; Invitrogen, Grand Island, NY, USA). Possible lipofuscin artifacts were quenched by treating slides with 3% Sudan Black B stain as described previously (36). Sections were rinsed in Dulbecco's PBS and coverslipped with VECTASHIELD Vibrance antifade mounting medium with or without 4′,6-diamidino-2-phenylindole (Vector Laboratories). Gallya's staining was performed as described (37). Briefly, slides were treated with 5% periodic acid for 5 min, washed with water, and incubated sequentially in silver iodide (1 min) and 0.5% acetic acid (10 min) solutions prior to being placed in developer solution (2.5% sodium carbonate, 0.1% ammonium nitrate, 0.1% silver nitrate, 1% tungstosilicic acid, 0.7% formaldehyde). Slides were treated with 0.5% acetic acid to stop the reaction, then incubated with 0.1% gold chloride and placed in 1% sodium thiosulfate. Following a final wash in water, slides were dehydrated and coverslipped. Congo red histology was performed as described (38). Briefly, 2.5 mM NaOH was added to a saturated sodium chloride-ethanol solution, and slides were incubated for 20 min. Subsequently, slides were incubated in 0.2% Congo red in alkaline alcoholic saturated sodium chloride solution for 30 min. Slides were rinsed through 3 changes of 100% ethanol, cleared through 3 changes of xylene, and coverslipped with Di-N-butyl phthalate in xylene (DPX; VWR, vwr.com).

#### Tissue Imaging and Quantification

Immuno-labeled sections were imaged using a Zeiss Axio Scan Z1 digital slide scanner at various magnification as indicated in each figure legend. Digital images of each slide and its sections were analyzed for threshold-defined pixel-positive area fraction using custom-designed image analysis software (Nearcyte, Zeiss). Values for all sections from the same mouse were averaged to represent a single value for that region in subsequent statistical analysis. For all analyses, an investigator blinded to the study conditions captured the hippocampi for each animal (*N* = 6, 8 section per animal).

Immunofluorescent labeled sections were imaged using a Nikon C2Plus Confocal Microscope. Representative images of the Tmem119 and Iba-1 microglia positive surfaces were capture using galvano-sequential scanning and 60x magnification was captured using the z-stack feature (1.2 AU pinhole, 50 nm pixel size) in NIS-Elements AR 5.11.01 software. For co-localization analysis, Mander's overlap coefficient was preferred over Pearson correlation coefficient, to avoid differences in fluorescence intensities between both antigens and the auto-background (39).

#### Sholl Analysis

To measure the astrocytic arborization we performed Sholl analysis of GFAP positive astrocytes using the Fiji plug-in for ImageJ (http://fiji.sc/Sholl_Analysis) following previously published analysis protocol (40). Briefly, individual cells with maximum projection were analyzed using fluorescence imaging (16-bit greyscale). Images were adjusted to the brightness and contrast threshold as indicated in the analysis software. A line was drawn manually from the soma of astrocytes to the end of its arbors to ensure maximum intersection points per cell. The radius of 50 μm was kept constant for each cell analyzed within the region of interest (ROI). From the tiled 2D images of the cortex the morphology of selected astrocytes was isolated. Sholl analysis software was used to retrieve the cellular metrics. The number of intersections from the output data were graphed using GraphPad Prism 8.03.

### Western Blotting

Tissues for Western blot analysis were prepared as previously described (41). Dissected hippocampi (HPC) were homogenized then sonicated in radio-immunoprecipitation assay (RIPA) buffer containing protease inhibitor cocktail (Sigma Aldrich) and phosphatase inhibitor cocktails I and II (Sigma Aldrich) and centrifuged at 40,000 x G for 30 min at 4°C. The supernatant was collected, and protein concentrations were determined by the BCA protein assay kit (Pierce, Rockford, IL). The remaining pellet was dissolved with 70% formic acid according to the wet tissue weight, and then neutralized with NaOH to analyze RIPA-soluble proteins. Equal amounts of proteins according to BCA (5 μg/well for soluble fraction, 1 μg/well for insoluble fraction) were loaded in each well of a 4–12% Bis-tris gels and transferred to a 0.2 μm pore size nitrocellulose membrane and immunoblotted with H150 (Santacruz Biotechnology, Dallas, TX), pSer396 tau (Anaspec, Fremont, CA), PHF1 (Fisher Scientific, Waltham, MA), AT180 (Fisher Scientific, Waltham, MA) and pSer199/202 tau (Anaspec, Fremont, CA) at 1:1,000-fold dilution. Band intensities at the 55 and 64 kDa range were quantified by densitometric analysis using AlphaEase software (Alpha Innoch, CA, USA) and normalized to the band intensity of GAPDH (for RIPA soluble fraction) and total protein (RIPA insoluble fraction).

### Multiplex Chemokine/Cytokine Assay

Snap-frozen right hippocampi from all the animals were homogenized in RIPA buffer (50 mM Tris, pH 7.5, 150 mM NaCl, 1 mM ethylene diamine tetra-acetic acid, 1% Triton X-100, protease inhibitor cocktail and phosphatase inhibitor cocktail I and II; Sigma). The protein concentration of each sample was measured using the Bradford protein assay (Bio-Rad Laboratories, Hercules, CA, USA) and adjusted to 4.5 mg/ml. The concentrations of Interleukin-6, C-C motif ligand 3 (CCL3), Interleukin 1 alpha (IL-1α), C-C motif ligand 5 (CCL5), Tumor Necrosis Factor-alpha (TNF-α), Interleukin 1 beta (IL-1β), Interleukin 10 (IL-10), Interleukin 13 (IL-13), Vascular endothelial growth factor (VEGF), KC, C-C motif ligand 11 (CCL11), Interferon-gamma (IFN-γ), and Interleukin 4 (IL-4) were measured using the mouse cytokine/chemokine panel (MILLIPLEX MAP kit; Millipore, Billerica, MA, USA) according to the manufacturer's protocol. In brief, the Bio-Plex Suspension Array System (Bio-Rad Laboratories) was calibrated using CAL2 with the high PMT setting of the Bio-Plex calibration kit, and standard sample preparation was performed according to the manufacturer's directions. The filter plate was pre-wetted with wash buffer and vacuum-filtered before adding standard, control or study samples to the appropriate wells. Mixed capture beads were then added to each well, and plates were incubated overnight at 4°C with shaking. After two washes, 25 μl of detection antibody was added to each well, incubated for 1 h at room temperature and then treated with 25 μl of streptavidin-phycoerythrin for 30 min at room temperature. The plate was washed twice, and 150 μl of the Bio-Plex sheath fluid assay buffer were added to each well and read using the Bio-Plex Suspension Array System software (Bio-Rad Laboratories) per the kit instructions. The concentration of each analyte was calculated according to the standard curve.

### Statistical Analysis

For each genotype (either rTg4510 or APP/PS1), statistical analyses were performed using Student's *t*-test followed by Fisher's least significant difference *post hoc* means comparison. For cytokine analysis, multiple *t*-test analysis was performed without assuming a consistent SD (df = *N*−2) and followed by *post-hoc* correction. Statistical analysis and graphs were generated using GraphPad Prism 8.03 software (GraphPad Software, La Jolla, CA, USA).

## Results

### Intracranial Injection of AAV9- CCL2 Induced Strong Expression of CCL2

**Figure 1:**
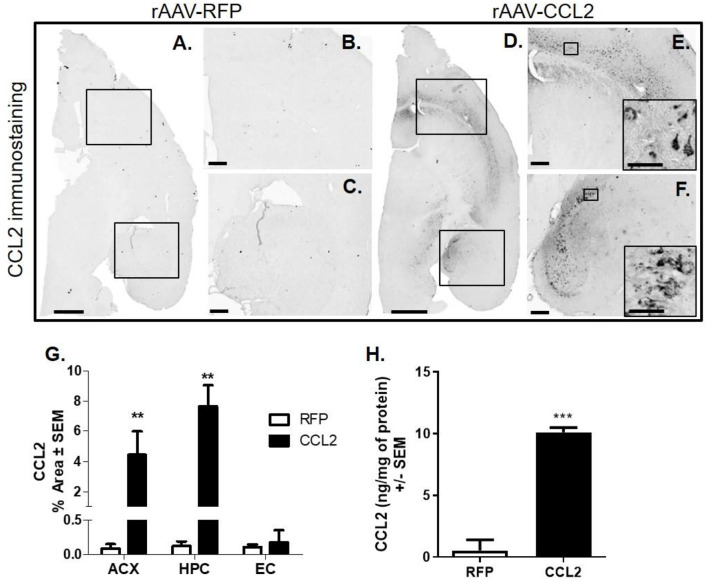
Intracranial injections of AAV9-RFP or AAV9-CCL2 in cortex and hippocampus in rTg4510 mice. Five-mo-old rTg4510 mice underwent intracranial bilateral injection of rAAV9-CCL2 (*n* = 6) or red fluorescent protein (RFP, *n* = 6) in both the hippocampus and the anterior cortex. Two months after the intracranial injections, brain tissue was collected. Micrographs represent the immunohistochemistry staining for CCL2 in RFP injected **(A–C)** and CCL2- injected animals **(D–F)**. Higher magnification shows cell localization of CCL2 (insets). **(G)** Percent positive area of distribution in anterior cortex (ACX, boxed area), hippocampus (DG and CA3, boxed area) and entorhinal cortex (EC) of injected animals (*n* = 6, **p < 0.01 and ***p < 0.001). **(H)** CCL2 levels were measured in the hippocampus of injected mice by multiplex assay and the results were normalized to the amount of protein (ng/mg of protein). Student's *t*-test were performed between RFP and CCL2 groups for each dependent variable and each brain region separately. Scale bar represents 1,000 μm in **(A,D)**, 200 μm in **(B,C,E,F)** and 20 μm in insets.

Overexpression of CCL2 was confirmed by immunohistochemical analysis (Figure 1). We demonstrate that rAAV9-CCL2 injected mice present increased CCL2 expression (Figure 1D) localized to injected areas i.e., cortex (Figure 1E) and hippocampus (Figure 1F). As expected, no CCL2 signal was detected in the rAAV9-RFP injected control mice (Figures 1A–C). Quantitation of area positively stained for CCL2 showed a significant increase of CCL2 expression in both cortex and hippocampus but not in synaptically connected areas such as the entorhinal cortex, when compared to rAAV-RFP (Figure 1G).

Quantitation of CCL2 protein levels in hippocampal tissue was performed by using the MILLIPLEX MAP Multi-plex assay kit (Figure 1H). Basal levels of CCL2 in rAAV9-RFP-injected animals were in the low range (0.52 ± 0.1 ng/mg of protein); however, as expected, CCL2 expression was significantly higher in the hippocampus of rAAV9-CCL2-injected animals (10.1 ± 0.2 ng/mg of protein) resulting in a 20-fold increase when compared to rAAV9-RFP-injected control animals (Figure 1H). The CCL2 levels measured here are in range to what was previously reported in the hippocampus of non-transgenic mice challenged with LPS (42, 43), suggesting that the ng range of CCL2 protein is required to maintain a prolonged inflammatory state during brain injury. We demonstrated that brain overexpression of CCL2 achieved through AAV9-transduciton was sustained 2 months after surgery.

### Overexpression of CCL2 in the Brain of rTg4510 Did Not Change Microglia Density but Resulted in Microglia Activation

**Figure 2:**
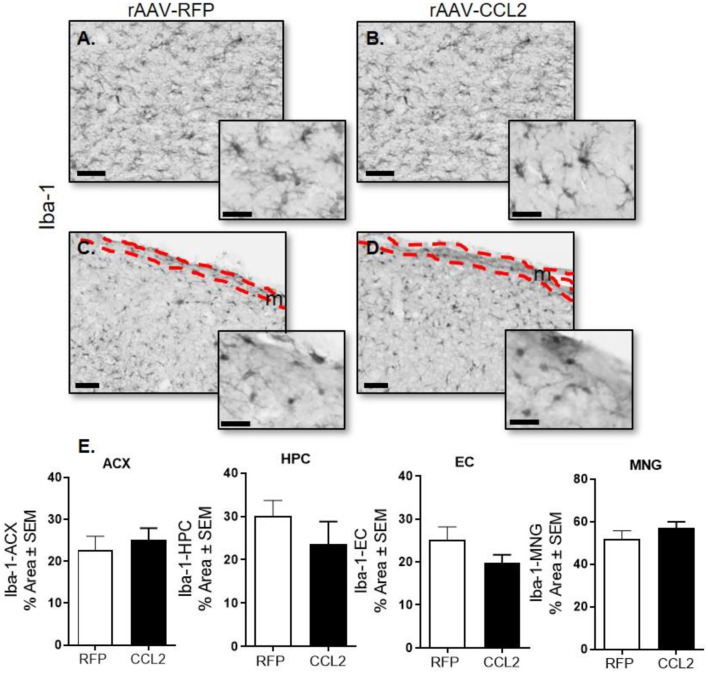
CCL2 overexpression does not affect microglia abundance in rTg4510. Micrograph representation of hippocampal area **(A,B)** and cortical area **(C,D)** stained for Iba-1 in RFP injected animals **(A,C)** and CCL2 injected animals **(B,D)**. Quantification of positive area stained is presented for anterior cortex (ACX), hippocampus (HPC) entorhinal cortex (EC) and meninges (MNG) for Iba-1 (mean ± S.E.M, *n* = 6) **(E)**. Student's *t*-test were prformed between RFP and CCL2 groups for each dependent variable and each brain region separately (*n* = 6). The scale bar represents 50 μm in panels and 20 μm in insets. Meningeal area is outlined in the cortical images (**C,D**, m).

We first assessed the effect of CCL2 overexpression on microglial abundance in rTg4510. Brain tissue was examined for microglial pan-marker Iba-1 immunoreactivity in rAAV9-RFP (Figures 2A,C) and rAAV9-CCL2 (Figures 2B,D) injected rTg4510 mice. Quantification analysis of Iba-1 positive area stained (Figure 2E) in cortical areas (Figure 2E, ACX, EC) and hippocampus (Figure 2E, HPC) revealed no difference in Iba-1 levels between CCL2 treated mice and RFP control mice. Similarly, levels of Iba-1^+^ positive meningeal macrophages remained similar between rAAV9-CCL2 mice and rAAV9-RFP expressing mice (Figures 2C,D,E, MNG).

**Figure 3:**
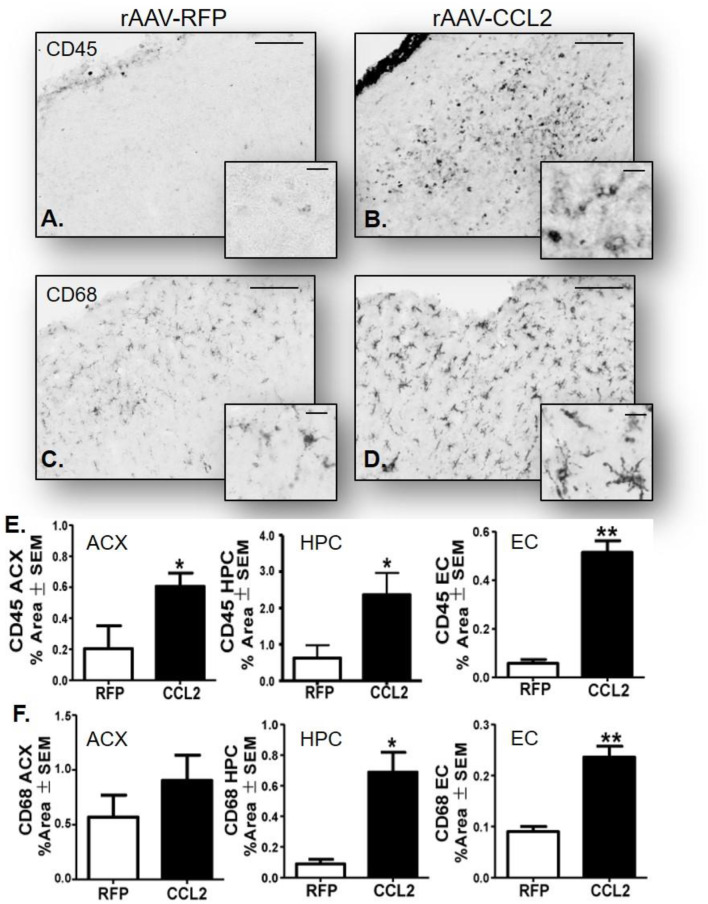
CCL2 overexpression induces microglia activation in rTg4510 brain. Micrograph representation of cortical area stained for CD45 and CD68 in RFP injected animals **(A,C)** and CCL2 injected animals **(B,D)**. Quantification of positive area stained is presented for anterior cortex (ACX), hippocampus (HPC) and entorhinal cortex (EC) for CD45 **(E)** and CD68 **(F)** (mean ± S.E.M, *n* = 6). Student's *t*-test were performed between RFP and CCL2 groups for each dependent variable and each brain region separately (*n* = 6, *p < 0.05, **p < 0.01). The scale bar represents 200 and 20 μm in panels and insets, respectively.

**Figure 4:**
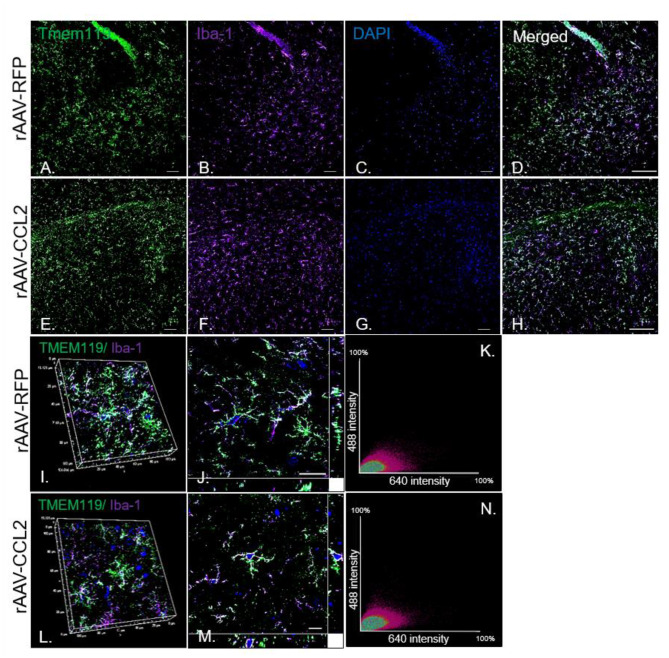
Tmem119 is stably expressed and co-localizes with Iba-1^+^ microglia in rTg4510 mice. Imaging of fluorescence labeled microglia with **(A,E)** Tmem119 (green) and **(B,F)** Iba-1 (purple) in the hippocampus of RFP- and CCL2-injected rTg4510 mice. Nuclei is stained with DAPI **(C,G)**. Merged images of Tmem119 and Iba1 immunoreactivity in brain sections from treated mice **(D,H)**. *N* = 6, 8 section per animal. Scale bar, 100 μm **(I,J,L,M)**. High magnification (60x) co-localization images utilizing z-stack image intensity of highly ramified Tmem119^+^ cells and Iba-1^+^ microglia. Scale bar, 10 μm. **(K,N)** The intensity correlation analysis represented by the scatter plots of the fluorescence intensities of Tmem119 (Alexa Fluor 488) and Iba-1 (Alexa Fluor 647) of confocal z-stacks. The degree of co-localization is estimated by Mander's overlap coefficient.

To further characterize the microglia activation profile following CCL2 overexpression, immunoreactivity of brain tissue for CD45 microglia was measured in rAAV9-RFP (Figure 3A) and rAAV9-CCL2 injected rTg4510 mice (Figure 3B). Quantitation of the positive area stained for CD45 using image analysis revealed significant increases in CD45 levels in anterior cortex (ACX), hippocampus (HPC) and entorhinal cortex (EC) of CCL2 overexpressing mice when compared to rAAV9-RFP control mice (Figure 3E). Similarly, immunohistochemical analysis for CD68 positive phagocytic microglia was performed (Figures 3C,D). Analysis of CD68 positive area revealed significantly increased levels of phagocytic microglia in the injected hippocampus (HPC), and connected regions, entorhinal cortex (EC), of rAAV9-CCL2 injected rTg4510 mice when compared to RFP injected littermates (Figure 3F). Although we found a trend toward increased CD68 levels in the anterior cortex of rAAV9-CCL2 when compared to RFP injected mice, the difference did not reach significance (Figure 3F). It is recognized that staining for CD45 or CD68 cannot unambiguously discriminate intrinsic microglia from recruited myeloid linage cells. Recently, it has been suggested that infiltrating macrophages in the brain can be discriminated based on their expression of Iba-1 and lack of co-localization with Tmem119, a highly expressed microglia-specific marker of resident microglia (44). Thus, to further dissect the microglia-specific response to CCL2, we performed immunofluorescence labeling between Iba-1 and Tmem119 and examined the relative expression of both markers (Figure 4). We observed no mean fluorescence intensity (MFI) difference between RFP and CCL2 in Tmem119^+^ and Iba-1^+^ microglia, suggesting that CCL2 did not affect the overall relative expression and/or microglia abundance (Figures 4A,B,E,F). High magnification imaging and z-stack image analyses demonstrated overlap of Tmem119^+^ with Iba-1^+^ relative intensity in both RFP- and CCL2-injected mice (Figures 4I,J,L,M). Further analysis of immunolocalization using Mander's overlap coefficient showed 0.89- and 0.91-pixel value average for RFP- and CCL2 -injected mice, respectively (Figures 4K,N), indicating that more of 90% of the Iba-1^+^ cells were enriched for Tmem119 immunoreactivity. Taken together, these findings suggest that albeit CCL2 did not increase the relative levels of Tmem119^+^/Iba-1^+^ intrinsic microglia, its overexpression had a vast impact on activation of CD45 and CD68 phagocytic microglia in the brain of rTg4510 mice.

### Overexpression of CCL2 in the Brain of rTg4510 Induced Astrocytic Dendritic Arborization

**Figure 5:**
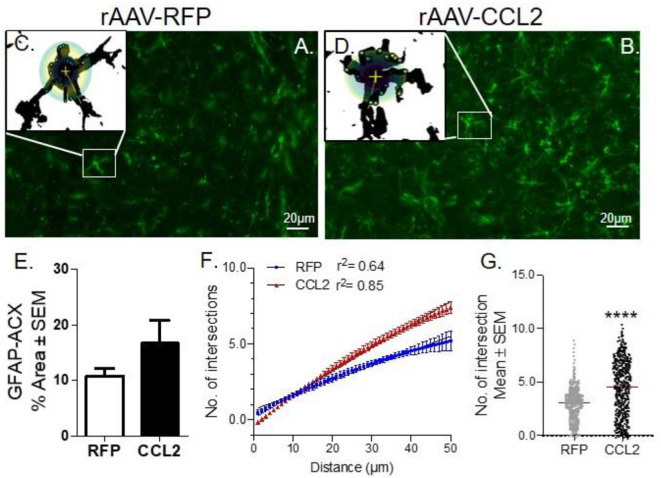
CCL2 induces astrocytic ramification in rTg4510 mice. **(A)** Images of fluorescence immunohistochemical stain of astrocytes (GFAP, green) in the cortices of rTg4510 mice following CCL2 and **(B)** RFP overexpression. Individual astrocytes were subjected to Sholl analysis (box inset). **(C,D)** Maximum intensity projection of each cell tiled micrographs extracting 2D images of astrocytes. Sholl-based metrics of arborization using a 50 μm radii area from the soma of each astrocyte measured the number of intersections. **(E)** Quantification of positive area stained for GFAP is presented for anterior cortex (ACX). **(F)** Sholl analysis retrieved curve-fitting and regression analysis of astrocytes within the region of interest, demonstrated induced astrocytic intersection in CCL2 overexpressing mice compared to the RFP mice. **(G)** Scattered plot of the number of astrocytic intersections in each group, Student *t*-test, Mann-Whitney unpaired parametric test, ****p < 0.0001.

Astrogliosis was measured via the immunoreactivity levels of glial fibrillary acidic protein (GFAP), an intermediate filament (IF) protein that is highly specific marker for the astrocytic cells in the brain (45) (Figure 5). Images from fluorescence immunohistochemical stain of astrocytes (GFAP, green) are shown following CCL2 expression in rTg4510 mice compared to RFP injected littermates (Figures 5A,B). Comparably to our observations on Iba-1, we found that GFAP immunoreactivity remained unchanged between groups, as represented by the quantification of positive area stained for GFAP in anterior cortex (Figure 5E, ACX). However, this finding does not account for the vast morphological changes of the astrocytes following CCL2 expression. This prompted us to perform Sholl analysis of the dendritic arbor complexity of GFAP positive astrocytes in the cortices of AAV9-CCL2 vs. AAV9-RFP mice (Figures 5B,D). We showed that astrocytic arborization in CCL2 mice was significantly increased compared to that of astrocytes from control mice (Figures 5A,C), as identified by the increased number of intersections per given astrocyte at a fixed distance from soma (Figures 5F,G). These results indicate that enhanced dendritic development occurred in astrocytes upon overexpression of CCL2 in rTg4510 mice.

### AAV9-Mediated CCL2 Overexpression Induced Changes in Levels of Cytokines Associated With Neuroinflammation

**Figure 6:**
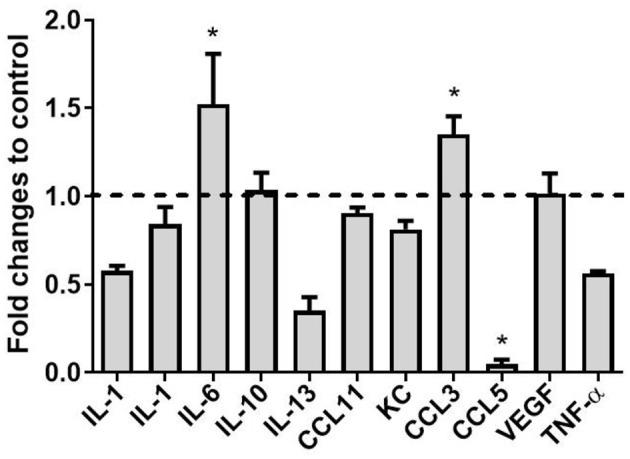
Cytokine levels following expression of CCL2 in rTg4510 mice. The concentrations of Interleukin 1 alpha (IL-1α), Interleukin 1 beta (IL-1β), Interleukin 6 (IL-6), Interleukin 10 (IL-10), Interleukin 13 (IL-13), C-C motif ligand 11 (CCL11), Keratinocyte chemoattractant (KC), C-C motif ligand 3 (CCL3), C-C motif ligand 5 (CCL5), Vascular endothelial growth factor (VEGF) and Tumor necrosis factor alpha (TNF-α) were measured using the mouse cytokine/chemokine panel (MILLIPLEX MAP kit; Millipore, Billerica, MA, USA) in CCL2 injected mice and RFP controls. (mean ± S.E.M, *n* = 6). Statistical analysis was performed using multiple *t*-test analysis (*p < 0.05) with alpha = 0.05. and without assuming a consistent SD (df = N−2) followed by *post-hoc* test.

We have previously reported the impact of CCL2 on microglia polarization in wild-type mice (23, 46). Therefore, we aimed to investigate the effect of CCL2 on expression levels of a range of cytokines in a tau animal model. Hippocampi from rAAV9-CCL2- and rAAV9-RFP -injected rTg4510 mice were homogenized and chemokines were measured using multiplex assay (Millipore, Billerica, MA). We observed 1.5-fold increases in the levels of IL-6 and CCL3 (Figure 6), in the hippocampi of CCL2- compared to RFP-injected mice (49.9 ± 1.9 vs. 18.0 ± 9.4 pg/ml and 4.5 ± 0.4 pg/ml vs. 3.3 ± 0.3 ng/ml, respectively). In addition, CCL5 levels were significantly decreased in response to CCL2 overexpression (0.4 ± 0.2 vs. 7.2 ± 3.6 ng/ml in CCL2-injected animals vs. RFP control animals, respectively). No significant changes were observed in, IL-1α, TNF-α, IL-1β, IL-10, IL-13, VGEF, KC, and CCL11 cerebral levels, while levels of IFN-γ and IL-4 were undetectable. Interestingly, low nanogram range of IL-12 levels were measured in RFP injected control mice (0.2 ± 0.1 ng/ml) but were undetectable in CCL2-injected animals (data not shown). We conclude that CCL2 overexpression in tau mice produced a unique pattern of inflammatory signals.

### Brain CCL2 Overexpression Aggravated Tau Pathology

**Figure 7:**
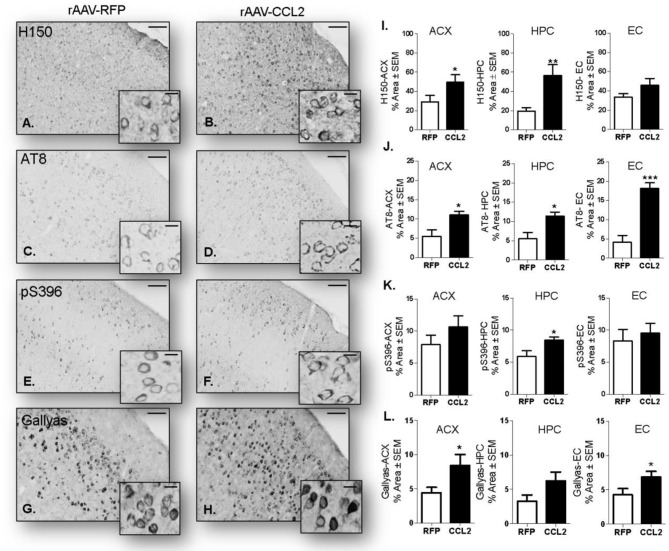
CCL2 overexpression induces tau accumulation in rTg4510 mice. Images of immunostaining in the cortical area for H150 tau **(A,B)**, p-tau AT8 **(C,D)** and pSer396 **(E,F)** as well as Gallyas staining (aggregated tau **G,H**) in mice injected with either rAAV9-RFP or rAAV9-CCL2. Quantification of positive area stained is shown in anterior cortex (ACX), hippocampus (HPC), entorhinal cortex (EC) for total tau H150 **(I)**, p-tau AT8 **(J)**, pSer396 **(K)** and Gallyas **(L)**. Scale bar represents 100 and 20 μm. Statistical analysis by Student *t*-test (*n* = 6, *p < 0.05, **p < 0.01, ***p < 0.001).

Next, we assessed the effect of CCL2 overexpression on tau pathology, including immunoreactivity of brain tissue for phosphorylated tau (p-tau) isoforms and neurofibrillary tangle burden, in rAAV9-CCL2 and rAAV9-RFP injected rTg4510 mice. Micrograph representation of immunostaining in anterior cortex (ACX) is shown for H150 (total tau (H150), Figures 7A,B), AT8 (pSer199/Thr205, Figures 7C,D), pSer396 (Figures 7E,F) as well as Gallyas staining (neurofibrillary tangles, Figures 7G,H). Quantitation of positive area stained revealed a significant increase in total tau levels in ACX and hippocampus (HPC) as measured by the H150 antibody (Figure 7I). Interestingly, AT8 p-tau levels were significantly increased in both injected regions (ACX/HPC) and synaptically connected entorhinal cortex (EC) of CCL2 mice (Figure 7J). The pSer396 tau levels were also significantly increased in HPC but not in ACX or EC of CCL2 mice (Figure 7K). Of note, we also observed a significant increase in Gallyas staining in the ACX and EC, while a trend toward increased tau burden was achieved in the HPC region (*p* = 0.08) of CCL2 mice when compared to RFP injected control mice (Figure 7L).

**Figure 8:**
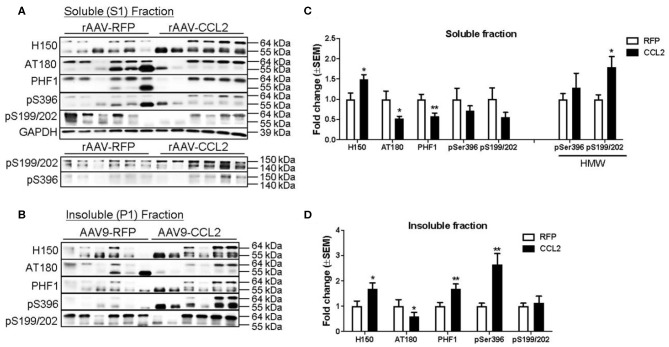
CCL2 overexpression worsens tau pathology in rTg4510 mice. Western blots analyses of total tau (H150), and p-tau (AT180, PHF1, pSer396, and pSer199/202) in the soluble hippocampal brain fraction **(A)** and insoluble fraction **(B)**. Band pixel densitometry values normalized to GAPDH and control mice for soluble fraction **(C)**, and to controls for insoluble fraction **(D)** (*n* = 6, *p < 0.05, **p < 0.01, Student *t*-test). Overall, CCL2 overexpression resulted in increased high molecular weight tau in soluble fractions together with increased intensity of phosphorylated epitopes in insoluble fractions.

Biochemical analysis was performed to distinguish between detergent soluble and insoluble tau levels in the brain. Western blots were analyzed for levels of total tau (H150), and p-tau epitopes (AT180, paired helical filaments 1: PHF-1, pSer199/202, and pSer396) in the soluble fraction (Figures 8A,C) and in the insoluble fraction (Figures 8B,D) of hippocampal brain extracts of injected mice. Band pixel densitometry values were normalized to GAPDH and then represented as the percentage of controls. We found a significant increase in total tau levels (H150, 55-64 kDa band) in the soluble fraction, while AT180 and PHF-1 p-tau levels were significantly decreased following CCL2 overexpression (Figure 8C). No changes were observed in pSer199/202 or pS396 p-tau levels in this fraction. However, we observed a significant increase in pSer199/202 but not pSer396 high molecular weight band (140–150 kDa) intensity in the soluble fraction (Figure 8C, HMW). No signal was detected for high molecular weight of H150, AT180, and PHF-1. Interestingly, our analysis revealed significant increases in insoluble total tau (H150), PHF-1 and pSer396 p-tau levels (55–64 kDa, Figure 8D), while no changes were observed in pSer199/202 or AT180 p-tau. Consistent with the worsening of tau pathology observed by immunostaining, western blot analysis revealed a shift in tau solubility following CCL2 overexpression, where an increase in total tau in all fractions reflected a decrease in soluble tau forms while increasing levels of certain insoluble tau forms in CCL2 mice.

**Figure 9:**
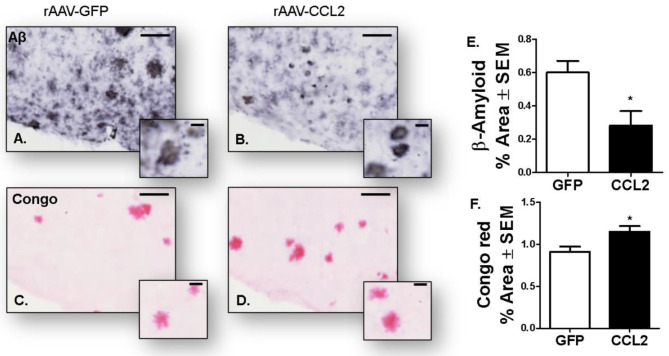
Amyloidosis following CCL2 overexpression in APP/PS1 mice. Micrograph representation of cortical area stained for amyloid beta (Aβ, **A,B**) and Congo red **(C,D)** in GFP injected animals **(A,C)** and CCL2 injected animals **(B,D)**. Quantification of positive area stained is presented for anterior cortex amyloid beta **(E)** and Congo red **(F)** (Avg ± S.E.M, *n* = 10). Statistical analysis was performed using Student's *t*-test (*n* = 6, *p < 0.05) followed by Fisher's PLSD multiple comparison test. Scale bar represents 200 μm and insets represent 20 μm.

Interestingly a similar shift from soluble to insoluble species was observed in CCL2-overexpressing APP/PS1 mice. Indeed, we observed a decrease in β-amyloid immuno-positive area in the cortex of APP/PS1 mice (Figures 9A,B,E), while an increase in amyloid plaque burden positive for Congo red was evident in the same region compared to RFP-injected APP/PS1 mice (Figures 9C,D,F).

## Discussion

This study shows for the first time a role of CCL2 overexpression in exacerbation of tau pathology in rTg4510 mouse model, associated with enhanced glial cells activation and changes in inflammatory markers. Indeed, aggravated tau pathology following CCL2 expression was observed as an overall increase in total tau levels in the hippocampus independently of soluble or insoluble fractions. Interestingly we observed a redistribution of some of the p-tau epitopes toward increased aggregation state. We detected a decrease in the soluble fraction of AT180 and PHF1 p-tau species while the same species were increased in the insoluble fraction. High molecular weight tau species were also increased together with increased Gallyas positive neurofibrillary tangles following CCL2 expression.

We also report that worsening of tau pathology was associated with microglia activation and inflammatory response. We have previously shown that CCL2 overexpression in the brain of non-transgenic mice specifically increased microglia polarization as well as induced extravasation of peripheral myeloid-derived cells (CD45 positive) (23). In the present study, unchanged immunoreactivity for Iba-1 microglia was found in CCL2 injected rTg4510 mice when compared to RFP-injected transgenic littermates, reflecting no difference in microglia abundance. However, we observed enhanced microglia activation determined by increased levels of CD45 and CD68 staining in CCL2 injected rTg4510 mice when compared to RFP-injected transgenic littermates. As CD45 and CD68 are common markers of both intrinsic microglia and recruited myeloid lineage cells, we further investigated the origin of the Iba-1^+^ cells in the present study, by immunofluorescence labeling with microglia specific marker, Tmem119 (44). We report that approximately 90% of the Tmem119^+^ cells overlapped with Iba-1^+^ cells across the study, suggesting that overexpression of CCL2 had no effect on the overall levels of Tmem119^+^/Iba-1^+^ resident microglia in rTg4510 mouse models. In addition, Sholl quantification of astrocytic complexity found significant increase in astrocytic arborization of CCL2 mice compared to control mice, suggestive of astrocyte activation. In a similar fashion to that observed in microglia, CCL2 overexpression in rTg4510 was not associated with increased astrocytic density but increased astrocytic dendritic development. Further future experiments are required to establish the role of CCL2 in astrocytic architechture and activation.

Previous findings from our laboratory and others have demonstrated microglia activation and inflammatory profile in response to pathological tau in rTg4510 mice (47), as indicated by high immunoreactivity of Iba-1, CD45 and GFAP (37, 41, 48, 49) when compared to age-matched non-transgenic littermates. This inflammatory profile seems to vary with age in rTg4510 (37, 48) and to be tau dependent as it was reversed by suppression of the tau transgene expression (48, 49). In a similar fashion, we and others have reported elevation of CD45 and CD68 microglia levels and increased pathology in rTg4510 mouse model following neuroinflammatory manipulations. Indeed, cerebral LPS injection resulted in increased CD45 and Ym1 immunoreactivity, which was associated with increased tau phosphorylation without changes in total tau levels or tau tangle formation. Similarly, mice deficient for fractalkine where characterized by increased CD45 and CD68 levels as well as worsening in tau pathology (50). In contrast, fractalkine overexpression was associated with decreased levels of CD45 microglia and tau pathology (51). In studies involving neuroinflammatory manipulations in mice model of proteinopathies, deciphering whether microglia activation is a cause, or a consequence of increased pathology remains challenging. To the same extend, the exact mechanism on how CCL2 contributes to tau pathology was not fully elaborated here. One of the limitations in our study is the ability to discriminate the effects of CCL2 on infiltration of peripheral cells and their subsequent effect on tau pathology. Indeed, BBB breakdown induced by disruption of adherents junctions in response to CCL2 has been evidenced (52) and could play a role in worsening of tau pathology by facilitating, for instance, leucocyte transmigration into the brain. We also can't exclude the possibility of a third and unrelated mechanism that could link CCL2 to induced tauopathy, while microglia activation is a by-product of tau pathology worsening. Together with the role of CCL2 described in the literature and our previous reported findings (23), our current findings suggest that CCL2 increased microglial and astrocytic activation following overexpression and was associated with worsening of tau pathology in rTg4510 mice.

To further assess the inflammatory profile associated with CCL2 overexpression in rTg4510, we measured a panel of different cytokines spanning along the inflammatory activation scale. Here, we report that increase in CCL2 was accompanied by increased levels of cytokines IL-6 and CCL3. These cytokines have been shown to be produced by glial cells *in vitro* (53) and *in vivo* (54). Therefore, we suggest that activated glial cells are the major source of the measured cytokine levels following CCL2 overexpression. Interestingly, the meningeal infiltrated T cells can also secrete cytokines and maintain the homeostatic cytokine profile in the brain (55). Although not measured in this study, our group has reported that impaired blood brain barrier and T-cell, red blood cell and immunoglobuline infiltration was evident in rTg4510 mouse model as early as 9 months of age (49). Despite as much as 60% of the meningeal area displaying Iba-1 immunoreactivity, we found that CCL2 did not increase the abundance and Iba-1^+^ positive immune cells in the meninges. This could be due to other underlying aberrant processes implicating different immune cells infiltration in this model as disscused above.

Additionally, we report that CCL2 overexpression resulted in increased IL-6 production in the hippocampus. Altered IL-6 expression is found in CSF and around amyloid plaques in the brain of Alzheimer's disease (AD) patients (56, 57). In particular, IL-6 stimulates the production of APP in neurons *in vitro* (58), while cultured glial cells stimulated with APP can trigger IL-6 production (53). Moreover, IL-6 also enhances neuronal damage induced by Aβ peptide in cultured rat cortical neurons (59). A direct link between increased IL-6 and tau phosphorylation was established *in vitro*. Indeed, IL-6 administration in rat neurons led to an increase in p-tau species but not total tau, through activation of JAK/STAT and MAPK-p38 (60). IL-6 and CCL2 can induce each other and cooperatively elicit chemoattraction and recruitment of activated immune cells in a positive feedback loop to maintain and amplify a proinflammatory-like profile (61–63). Further analysis would be necessary to consider whether a similar pathway was involved in the worsening of tau pathology observed in the present study.

We also report that CCL2 overexpression in rTg4510 was associated with an increase in CCL3 production in the hippocampus. CCL3 is a chemokine involved in chemotaxis and has been shown to contribute to T cell recruitment to the brain (64, 65). In accordance with our data, increased CCL3 production by microglial cells was observed in a different model of tauopathy, THY-Tau22 mice (54). The authors report that CCL3 induced the recruitment of T-cells into the hippocampus through chemotaxis, which promoted tau pathology in this model. T-cell infiltration has been previously demonstrated in older (12 months old) rTg4510 mice and was associated with a leaky blood-brain barrier (49). Albeit not investigated in this study, the possibility of a leaky BBB at this age together with the induction of IL-6 and chemotactic CCL3 and CCL2 signaling could suggest that aggravated tau pathology is a result of downstream events resulting from a plethora of infiltrated cells, including T-cell leucocytes.

More investigation of the role of CCL2 on tau pathology is needed to further understand the possible relationship between CCL2 signaling and tau burden. In fact, the role of CCL2 in AD has been studied more extensively in amyloid β animal models of AD than in tauopathies and have led to contradictory findings. Indeed, genetic manipulations of CCL2 in a mouse model of amyloidosis led to the worsening of Aβ pathology with both overexpression and deficiency in CCL2. Moreover, overexpression of CCL2 driven by GFAP promoter in APP/CCL2 mice enhanced amyloidosis, increased microglial Iba-1immunoreactivity, and decreased cognition (16, 17) while APP/PS1/CCL2 null mice also displayed increased levels of Aβ oligomers and worsening of cognitive dysfunction (18). The authors hypothesized that two different mechanisms could be involved, namely overexpression of CCL2 might promote Aβ assembly and microglial activation, whereas a deficiency in CCL2 would impair phagocytosis and clearance of Aβ (18). Our present findings support an increase in amyloidosis following CCL2 overexpression in the brain of APP/PS1 mice. Moreover, we found a decrease in soluble β-amyloid, that was redistributed toward amyloid plaque burden following 4 months of CCL2 expression, similar to what was observed with tau pathology in rTg4510 mice. In agreement, a disruption of CCL2 signaling through deficiency in CCL2 receptor in APP/CCR2 null mice precipitates Aβ accumulation by decreasing Aβ clearance in APP mice (19). However, a recent report showed improved cognition and decreased amyloidosis in 5xFAD/CCL2 null mice, another amyloid mouse model (20). Decrease in pathology was associated with a decrease in GFAP immunoreactivity in these mice, while control CCL2 null mice displayed an increase in IL-1β and GFAP immunoreactivity compared to non-transgenic mice. Altogether, these reports suggest that CCL2 action and signaling depend on the inflammatory milieu of the animal models employed and could lead to beneficial as well as detrimental responses. Another interesting report suggests that a gradient in CCL2 is required for its chemotactic capacity [reviewed in (66)]. Thus, it is possible that basal levels of CCL2 are required for brain homeostasis and therefore deficiency or overexpression would lead to detrimental effects on neuroinflammation. These dual effects of microglia are in accordance with the hypothesis of a dual peak of microglia activation in Alzheimer's trajectory recently advanced, which describes an early protective peak and a later pro-inflammatory peak in Alzheimer's disease pathophysiology (67). The contradictory results observed in AD models could be consequently explained by different stages of the diseases at the time of the experiment. Future studies involving neuroinflammation modulation should consider testing different time points of the disease stage. Identifying molecules with a pivotal role in determining the detrimental or beneficial effects of microglia is also important.

Altogether our results demonstrate an increase in tau accumulation following CCL2 overexpression in the rTg4510 mouse model of tauopathy together with glia activation, ultimately changing the neuroinflammation milieu. Given the contradictory results observed in mouse models of amyloidosis, more studies involving the role of CCL2 signaling concerning tau pathology are needed. Moreover, the degree of pathological insult and stage of the disease; i.e., Braak staging should be taken into consideration for translational relevance. Nevertheless, this is the first report linking directly CCL2 overexpression with increased tau pathology, providing important new anti-inflammatory avenues for future therapeutic intervention in AD and related tauopathies.

## Data Availability Statement

The datasets generated for this study are available on request to the corresponding author.

## Ethics Statement

All animal testing procedures were approved by the Institutional Animal Care and Use Committee of the University of South Florida and were performed in accordance with the eighth edition of the Guide for the Care and Use of Laboratory Animals, published by the National Academy of Science, the National Academies Press, Washington, DC (2011).

## Author Contributions

FZ and AK performed immunohistochemical analysis for amyloid β levels and Congo red histology to measure amyloid plaque burden in mouse tissue. ZQ performed biochemical assay and analysis. JH, DC, and DL performed immunohistochemistry assay and data analysis. M-LS and PR-R performed confocal imaging and analysis. KN designed and assisted DL in viral production approach. AJ-A and M-LS interpreted the data and wrote the manuscript. MG bred the mice and performed genotyping. DM, MG, and M-LS designed and conceptualized the study.

## Conflict of Interest

The authors declare that the research was conducted in the absence of any commercial or financial relationships that could be construed as a potential conflict of interest.
